# Improving Pharmacy Students’ Clinical Knowledge on Providing Care for Patients Belonging to the LGBTQ+ Community

**DOI:** 10.3390/pharmacy10040070

**Published:** 2022-06-24

**Authors:** Tyler C. Melton, William T. Johnson, Brittany Tipton, Kelsea G. Aragon, Calvin C. Daniels, Chelsea Phillips Renfro

**Affiliations:** 1 University of Tennessee Health Science Center, College of Pharmacy, Memphis, TN 38103, USA; wjohns34@uthsc.edu (W.T.J.III); btipton7@uthsc.edu (B.T.); crenfro@uthsc.edu (C.P.R.); 2 College of Pharmacy, University of New Mexico, Albuquerque, NM 87106, USA; kelsgall@salud.unm.edu; 3 St. Jude Children’s Research Hospital, Memphis, TN 38105, USA; calvin.daniels@stjude.org

**Keywords:** community pharmacy services, sexual and gender minorities, LGBTQ, health disparity, pharmacy education, curriculum

## Abstract

A 12-question survey instrument was developed, pilot-tested, and administered to 191 pharmacy students in their first professional year after engaging in a learning activity focusing on topics across five categories with clinical relevance to providing care to the LGBTQ+ community. A paired student *t*-test was performed on survey tool pre-test and post-test survey responses, with *p* < 0.05 considered significant. A total of 183 usable pre-test and post-test survey responses were received. Statistically significant differences between the pre-test and post-test correct responses were observed for scenarios involving proper pronoun use, hormone therapy (HT) counseling, immunization best practices, and communication hesitancy. The greatest knowledge change was reported in the categories of immunization best practices (48.9%), HT counseling (33.6%), and pronoun use (22.8%). Active learning assignments are effective teaching strategies to promote clinical knowledge in providing inclusive and culturally competent care to LGBTQ+ patients. Clinical topic areas including proper pronoun use, HT counseling, immunization best practices, privacy, risk awareness, and communication hesitancy are effective curricula additions for pharmacy colleges to advance inclusive curricula concerning providing care to the LGBTQ+ community.

## 1. Introduction

The community of patients identifying as lesbian, gay, bisexual, transgender, questioning/queer, and other identities (LGBTQ+) have historically been considered an underserved population in the American healthcare system [1,2]. Patients who are LGBTQ+ are less likely to receive proper healthcare due to both stigma and a lack of knowledge from providers, including pharmacists [1,3,4]. Some barriers to seeking healthcare for the LGBTQ+ community include the patient’s comfort in accessing healthcare and the medical provider’s education on providing inclusive healthcare [1,2].

Exploring how pharmacists can address health disparities facing the LGBTQ+ community, pharmacy colleges have begun to investigate how the LGBTQ+ community is served by pharmacists [5,6]. However, there is still little information about this topic. Only 33% of the United States’ pharmacy colleges reported topics related to trans or nonbinary individuals that were included during the didactic coursework, and 39% of colleges without such topics did not plan on making any additions [5]. Of the colleges that include content related to the care of patients that are transgender, many include trainings on cultural sensitivity, lectures, discussions, or case-based activities. Other additions included literature reviews, games, debates regarding the risks versus benefits of hormone therapy, implementing objective structured clinical examinations (OSCEs), and advanced pharmacy practice experiences (APPEs) specific to LGBTQ+ populations [5]. Of those that did not plan to include topics related to gender dysphoria, most colleges reported that their main obstacle was a lack of qualified faculty for instruction, as well as not having enough time to make additions to their curriculum [5]. Furthermore, 32% of curriculum deans self-responded to Mandap et al. that the inclusion of LGBTQ+ topics at their college was “very poor” [6].

The University of California at San Francisco’s lecture series on LGBTQ+ topics attracts more than 250 healthcare professionals each year, showing that healthcare providers want to be educated on these topics [7]. Implementing LGBTQ+ topics into pharmacy curricula offers the potential to bridge gaps in this patient population and offer insight into where improvements can be made. Programs that add topics and sessions related to disparities facing LGBTQ+ patients increase the inclusivity of their curriculum, foster a better understanding of cultural differences among students, and encourage innovation in the care of LGBTQ+ patients [5,6,7].

The expansion of pharmacy curricula to include LGBTQ+ topics provides an opportunity to operationalize non-traditional teaching strategies, such as active learning, in pharmacy colleges. Active learning strategies can be used to teach concepts that are difficult to grasp through student engagement in activities, allowing for the application of concepts more effectively compared to traditional lectures [8,9]. To meet the need for additional inclusive curricula within the pharmacy program, an active learning session centered on LGBTQ+ topics and clinical considerations was developed and implemented into the first professional year pharmacy curriculum to promote the development of clinical knowledge and skills specific to LGBTQ+ patients. The purpose of this study was to assess first-year student pharmacists’ knowledge before and after a single active learning session on clinical considerations and counseling points relevant to patients belonging to the LGBTQ+ community. To aid readers, a glossary of terms based on the definitions used by the Human Rights Council [10] is provided in Supplementary Table S1. 

## 2. Materials and Methods

### 2.1. Survey Design

A pre- and post-activity survey was designed to measure the impact of the active learning session on clinical considerations and counseling points relevant to patients belonging to the LGBTQ+ community. This learning activity was implemented in a required skills-based course during the spring semester of the first professional year at a college of pharmacy in the United States. The central hypothesis was that the active learning session would improve students’ clinical knowledge in providing care to members of the LGBTQ+ community.

The learning activity utilized the think–pair–share strategy and focused on topics with clinical relevance concerning the LGBTQ+ community [11,12,13]. These topics included: pronoun use; hormone therapy counseling; HIV and immunization best practices; communication hesitancy; privacy; patient inclusivity; and risk awareness. Prior to the in-class active learning session, the students were provided readings on effective communication and understanding the health needs for patients in the LGBTQ+ community and were administered the pre-test via a web-based software platform (QuestionPro, 2020) [14]. During class, two case scenarios (Table 1) were provided to students as part of an active learning exercise where students divided into small groups reviewed case scenarios and engaged in discussion questions facilitated by teaching assistants (TAs). Afterwards, class discussion and the reviewing of case solutions were facilitated by a faculty member and a representative from the campus’ LGBTQ+ outreach and inclusivity organization. Following the conclusion of the class, the students were administered the post-test survey. The assessment was pilot-tested with student teaching assistants and reviewed by pharmacy faculty prior to administration. Student knowledge was assessed using a pre-/post-survey administered to 191 first professional year students via a web-based software platform (QuestionPro, 2020). The assessment consisted of 12 questions with 9 multiple-choice and three true/false questions spanning the knowledge categories of pronoun use, hormone therapy counseling, HIV and immunization best practices, communication hesitancy, patient inclusivity, patient privacy, and risk awareness. This study was approved by the institution’s Institutional Review Board (IRB).

### 2.2. Data Analysis

The analyses were run in R version 1.0.136 (RStudio Team 2020). Each question’s pre- and post-survey results were analyzed via a paired student *t*-test, with *p* < 0.05 being considered significant. To maintain paired results, missing responses were removed from the analysis using pair-wise elimination. To determine the percent increase in correct responses between the pre- and post-test surveys, the number of students answering correctly during the pre-test was isolated and compared with the number of students answering correctly during the post-test. The response rates for each question’s available answer choices were broken down for both the pre- and post-tests to better visualize any patterns in the students’ responses, with correct responses, knowledge categories, correct response rates, and percent increases in correct responses being reported in Table 2.

## 3. Results

A total of 183 student responses were obtained, resulting in a 95.8% response rate for the pre- and post-surveys. Statistically significant differences between the pre- and post-test responses assessing proper pronoun use, HT counseling, HIV and immunization best practices, and communication hesitancy were reported (Table 2).

No statistically significant differences were reported between the pre- and post-test questions involving patient inclusivity (*p* = 0.06), matters related to privacy (*p* = 0.57), or risk awareness (*p* = 1.0). Three categories represent the largest increase in the average percent change for correct responses between the pre- and post-test multiple choice questions. These categories included HIV and immunization best practices (two survey questions: 48.9% increase), HT counseling (three survey questions: 33.6% increase), and pronoun use (two survey questions: 22.8% increase). The true or false questions had the largest increase in terms of percent change in the correct responses for the HIV and immunization best practices category (180% increase, or 1.8 times higher).

## 4. Discussion

Providing an inclusive pharmacy curriculum is paramount to training future pharmacists to deliver culturally competent and effective care to marginalized and often overlooked patient populations, such as the LGBTQ+ community [15]. This study assessed first-year pharmacy students’ knowledge on clinical considerations and counseling points relevant to patients belonging to the LGBTQ+ community before and after the delivery of a focused learning activity.

Proper pronoun use for transgender patients was a clinical knowledge focus area included in the learning activity. Both multiple choice questions relating to proper pronoun use reported statistically significant differences in the correct responses between the pre- and post-tests. Engaging students in an active learning exercise including proper pronoun usage allowed students to understand how to appropriately collect and address patients using preferred pronouns. The results from this active learning section reiterate the importance of providing pharmacy curricula that normalize providing gender-affirming care and align with clinical guidance and patient care guidelines to provide care for transgender and gender-diverse patients [15,16,17,18,19,20,21,22].

Effective testosterone and HT counseling was another clinical knowledge focus area in the learning activity. Bishop conducted a review of pharmacotherapy considerations in the management of transgender patients in 2015 and discussed the importance of pharmacists understanding the implications of hormone therapy for patients with gender dysphoria, as clinical decisions are influenced by health data which may be affected by a patient’s designated sex at birth [2]. Integrating effective testosterone and HT counseling points and transgender-related patient care into pharmacy curricula equips students with the clinical knowledge necessary to provide inclusive patient care [22]. While the majority of students answered pre-test questions correctly for this category, the differences between the results were statistically significant, indicating that students gained knowledge in this clinical focus area.

The think–pair–share strategy was used in the active learning exercise to allow students’ learning to become visible through the application of LGBTQ+ clinical knowledge in case scenarios. This active engagement in the subject matter possibly explains the highest percent increase across the three categories of pronoun use (22.8% increase), HT counseling (33.6% increase), and HIV and immunization practices (48.9% increase). Additionally, the learning activity proved useful in promoting students’ understanding of the stigma surrounding HIV and in providing appropriate immunization best practices. However, the pre-test responses may have been skewed due to the students’ stigma surrounding HIV exposure, the knowledge deficit, or the wording of the question [23,24].

Communication hesitancy surrounding patient–provider interactions, awareness of the risk of suicide, patient inclusivity, and the importance of privacy were topics included in the active learning exercise. Providing a context for students to understand these topic areas was important for students to develop and apply cultural competency in care [4,15,25]. The majority of participants correctly answered the privacy (68.7%) and communication hesitancy (70.8%) pre-test questions, indicating that the learning activity robustly activated prior knowledge. While most of the students answered the patient inclusivity question correctly, there was little change in the correct answer choice selection among the students who answered incorrectly, possibly indicating that more than one answer choice was perceived as correct.

The study results indicate that students have an appropriate level of understanding for the categories of inclusivity and awareness based on the pre- and post-test assessments. In contrast, the students experienced a change in knowledge for proper pronoun use, HT counseling, and HIV and immunization best practices. While the students displayed an appropriate baseline of knowledge for some categories and a knowledge gain in others, the learning activity was effective in providing knowledge critical to developing clinical skills and expertise appropriate to LGBTQ+ patients. Future applications of the learning activity can be improved. To further enhance this activity, future endeavors will aim to broaden the scope of the LGBTQ+ patient population covered in the activity; expand proper pronoun use to include the breadth of gender expression (i.e., non-binary, agender, genderfluid, etc.); collect student discussion in small groups; and, lastly, maintain the continued training of faculty to facilitate this learning through interdisciplinary collaboration and the involvement of LGBTQ+ organizations.

This learning activity was successfully implemented through the collaboration of TAs, faculty, and peers belonging to the LGBTQ+ community. The lack of qualified faculty for instruction in LGBTQ+ clinical considerations required the involvement and input from peers belonging to the LGBTQ+ community throughout the development of case-based discussions. As LGBTQ+ representation was a unique component of the learning activity, future reiterations will include feedback on how students plan to use this knowledge to provide care to patients belonging to the LGBTQ+ community during their IPPE and APPE rotations, as well as post-graduation. Additionally, a goal for the future research direction is to include an assessment of long-term LGBTQ+ clinical knowledge gain prior to the beginning of the fourth professional year (P4), when students begin Advance Pharmacy Practice Experiences (APPE).

## 5. Limitations

There are several limitations of this study. Student demographics were not collected at the time of the learning activity delivery. While this does not change the aggregate data analysis, individual demographics would be beneficial for a correlation analysis. Additionally, the post-test survey was administered immediately following the learning activity, which might result in short-term knowledge being assessed rather than lasting knowledge.

## 6. Conclusions

This study serves as an example to advance inclusive pharmacy curricula to focus on health disparities facing LGBTQ+ patients by providing relevant clinical considerations and counseling points. Pharmacy colleges must continue to revise and advance curricula to incorporate the clinical skills and cultural competency necessary to provide inclusive and effective care for LGBTQ+ patients. Learning activities addressing proper pronoun use, HT counseling, HIV and immunization best practices, communication hesitancy, patient inclusivity, patient privacy, and risk awareness are effective ways for pharmacy colleges to meet students’ and societal expectations for the incorporation of inclusive and relevant LGBTQ+ training.

## Figures and Tables

**Table 1:** Patient Case Scenarios.

	Case Scenario	Discussion Questions
Case #1	KG, a 27 year-old male (AFAB), walks into your pharmacy and requests education on how to give a subcutaneous testosterone shot. Upon questioning, he has a recently filled testosterone cypionate 200mg/10mL vial from your pharmacy but reports he did not receive syringes to administer it. His dose is testosterone 40mg subcutaneously once every week. You look up which syringes to use with testosterone cypionate and determine that the patient needs both a 25 gauge ½ in, 1cc syringe to administer testosterone as well as a 18 gauge syringe to draw the testosterone up. He has no known allergies or patient health conditions.	You know the patient is designated female at birth based on your pharmacy management system. You don’t want to make any assumptions and want to make the patient feel comfortable. What should your first interaction with this patient be? The patient asks how to dispose of the used needles. What do you tell him? KG states the testosterone was quite expensive. Upon examining the patient’s pharmacy management system, you find the patient does have insurance on file but under a different name. How would you approach this?
Case #2	VA, a 72 year-old male comes to your pharmacy to pick up his prescription for Biktarvy. He has never filled Biktarvy late and is one of your most adherent customers. You notice that he has not received his flu vaccine. You offer to administer the flu vaccine today and the patient agrees.	Knowing this patient is on Biktarvy are there any precautions you would take when administering a flu vaccine? What other vaccines should you ask VA if they have received?

AFAB = assigned female at birth.

**Table 2:** Survey questions and the percent change in correct answer responses.

Question	Correct Answer	Knowledge Category	Pre-Test Correct, n (%)	Post-Test Correct, n (%)	* Percent Increase in Correct Response, %	*p*-Value
1. When counseling a transgender patient, if you are unsure about which pronouns they use (he/him/his, she/her/hers, they/them/ theirs, etc.), what should you do?	Ask patient which pronouns they use.	Pronoun use	126 (69.2%)	172 (94.5%)	36.5%	<0.001
2. A transgender man approaches you at the pharmacy and asks you what needles he needs to use to inject his testosterone. You check the EHR to see by which route his doctor wrote to inject the testosterone. The prescription reads “Testosterone cypionate, inject into the muscle every week as directed.” What should you counsel him to do when injecting?	Draw up the testosterone with an 18G needle and use a 23G needle to inject.	HT Counseling	102 (55.7%)	146 (80.2%)	43.1%	<0.001
3. When giving a patient living with HIV an immunization, what should you tell them?	You use gloves with all your patients to protect them from pathogens you may have on your hands.	HIV and Immunization Best Practices	93 (50.8%)	160 (87.4%)	72.0%	<0.001
4. When counseling a patient on their hormone therapies, which of the following are best practices?	Use a private consult room to avoid outing the patient in a public place and avoid misgendering them.	HT Counseling	113 (61.7%)	157 (85.8%)	38.9%	<0.001
5. Which of these is the best way to make it apparent that your pharmacy is LGBTQ+ inclusive?	Have materials and signs that explicitly state that your pharmacy does not discriminate on the basis of race, gender, or sexual orientation, and include images of same-sex couples in education materials	Patient Inclusivity	122 (67.0%)	134 (73.2%)	9.8%	0.06
6. A woman (DMAB) designated male at birth) asks you to recommend hormone therapy. What are some factors you should take into consideration?	Because she was assigned male at birth, her insurance has the wrong gender and name on file. They will likely not cover hormone replacement therapy at first, and you may need to help her fix this issue with her insurance.	HT Counseling	112 (61.5%)	133 (72.7%)	18.8%	0.004
7. MC was assigned male at birth but now identifies as a woman who uses she/her/hers pronouns. How should you refer to her?	Refer to MC as a woman and use feminine pronouns (she/her/hers)	Pronoun Use	144 (78.7%)	157 (85.8%)	9.0%	0.03
8. Which of the following situation(s) may unintentionally “out” an LGBTQ+ patient/colleague who approaches your consultation window by themselves?	Discussing their sexual orientation in the waiting room where others can hear.	Privacy	127 (69.4%)	131 (71.6%)	3.2%	0.573
9. A patient living with HIV asks if he should receive a flu shot. What do you tell him?	He should receive the flu shot because people who are living with HIV are at greater risk of having complications due to flu	HIV and Immunization Best Practices	128 (69.9%)	161 (88.0%)	25.8%	<0.001
10. True or False Question: LGBTQ+ patients avoid accessing healthcare due to difficulty communicating with providers.	True	Communication Hesitancy	163 (89.1%)	176 (96.2%)	8.0%	0.003
11. True or False Question: There is an association between being an LGBTQ+ adolescent and suicide.	True	Risk Awareness	177 (95.7%)	178 (96.2)	0.6%	1.00
12. True or False Question: When vaccinating a patient living with HIV, it is important you take extra preventative measures to protect yourself from HIV exposure.	False	HIV and Immunization Best Practices	50 (27.6%)	140 (75.7%)	180%	<0.001

* Percent increase in correct responses was calculated using $\left[\frac{pretest\;\%\;correct-posttest\;\%\;correct}{pretest\;\%\;correct}\right]\times 100$.

## Data Availability

The data presented in this study are available upon request from the corresponding author at tmelto11@uthsc.edu. The data are not publicly available due to privacy concerns.

## Supplementary Materials

The following supporting information can be downloaded at: https://www.mdpi.com/article/10.3390/pharmacy10040070/s1, Table S1: Glossary of Terms.
