# Supplementary material for: Improving Pharmacy Students’ Clinical Knowledge on Providing Care for Patients Belonging to the LGBTQ+ Community
Source: Pharmacy (Basel). 2022 Jun 24;10(4):70. doi: 10.3390/pharmacy10040070 (PMC9326589; doi:10.3390/pharmacy10040070)
Supplement: Supplementary file 1 [file pharmacy-10-00070-s001.zip › new-pharmacy-1753490-supplementary.pdf]

pharmacy

Improving Pharmacy Students' Clinical Knowledge on Providing Care for Patients Belonging to The LGBTQ+ Community

Tyler C. Melton [1,*], William T. Johnson III [1], Brittany Tipton [1], Kelsea G. Aragon [2], Calvin C. Daniels [3] and Chelsea Phillips Renfro [1]

Table S1. Glossary of Terms.

Term	Human Rights Council Definitions [13]
Bisexual	A person emotionally, romantically or sexually attracted to more than one sex, gender or gender identity though not necessarily simultaneously, in the same way or to the same degree. Sometimes used interchangeably with pansexual.
Gay	A person who is emotionally, romantically or sexually attracted to members of the same gender. Men, women and non-binary people may use this term to describe themselves.
Gender Identity	One's innermost concept of self as male, female, a blend of both or neither – how individuals perceive themselves and what they call themselves. One's gender identity can be the same or different from their sex assigned at birth.
Gender Dysphoria	Clinically significant distress caused when a person's assigned birth gender is not the same as the one with which they identify.
Gender Nonbinary	Gender which does not fit as male or female.
Lesbian	A woman who is emotionally, romantically or sexually attracted to other women. Women and non-binary people may use this term to describe themselves.
LGBTQ	An acronym for "lesbian, gay, bisexual, transgender and queer."
Queer	A term people often use to express a spectrum of identities and orientations that are counter to the mainstream. Queer is often used as a catch-all to include many people, including those who do not identify as exclusively straight and/or folks who have non-binary or gender-expansive identities. This term was previously used as a slur, but has been reclaimed by many parts of the LGBTQ movement.
Sex	Designated based on external genitalia
Transgender	An umbrella term for people whose gender identity and/or expression is different from cultural expectations based on the sex they were assigned at birth. Being transgender does not imply any specific sexual orientation. Therefore, transgender people may identify as straight, gay, lesbian, bisexual, etc.

13. Lyman, F.T. The responsive classroom discussion: The inclusion of all students. *Mainstreaming Dig.* **1981**, *109*, 113.
